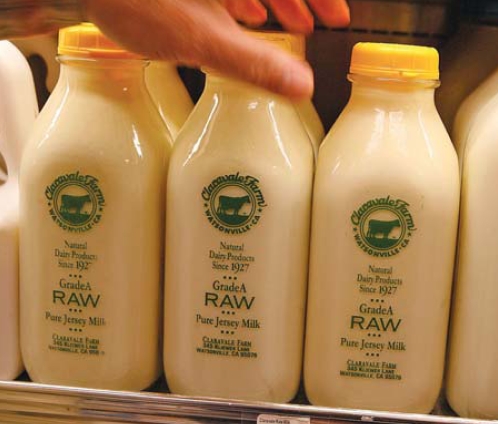# Does One Size Fit All?: Small Farms and U.S. Meat Regulations

**DOI:** 10.1289/ehp.116-a528

**Published:** 2008-12

**Authors:** David A. Taylor

Nationwide, the demand for locally produced food is growing dramatically. The U.S. Department of Agriculture (USDA) estimates the number of active farmers’ markets to have more than doubled since 1994, to almost 4,700. Community-supported farms, where customers sign up for a weekly allotment of meat, dairy, and produce through the season, have grown to nearly 1,500 nationwide. More than 2,000 school districts in 40 states work with local farms to obtain produce for student lunches, according to the National Farm to School Program based at Occidental College in Los Angeles. Amy Lanou, an assistant professor of health and wellness at the University of North Carolina at Asheville, says meat producers in her area can’t keep up with demand even though their product costs $1 more per pound than meat in the supermarket.

Much of the popularity of local farm products lies in their perceived benefits to consumer and environmental health. For meat products, reduced transport time between farm, slaughterhouse, and market means less opportunity for spoilage and hence less need for preservatives. Many small producers state a commitment to use fewer agricultural chemicals and antibiotics; this is possible because lower housing densities for pastured animals (compared with confined livestock) and a mixed-grass diet (compared with a high-grain diet) tend to reduce animal diseases. Benefits to the environment can include more sustainable long-term land management and less fossil fuel consumption getting products from farm to consumer—practices supported by the 2008 Farm Bill with the creation of the Conservation Stewardship Program.

Yet some small farm owners and advocates insist that the U.S. system for food inspection and safety—particularly in meat and poultry production—exacerbates an increasing centralization of American farming, squeezing small farms economically and hampering the local food movement. Moreover, they claim, the Hazard Analysis and Critical Control Point (or HACCP) plans required by the USDA of meat producers are skewed against small farms. Instead of the current mode of federal inspection and risk management, small-scale farmers and farm advocates believe rules should be based on independently measurable standards of sanitation and quality, with sensitivity to scale of the operation being assessed.

## The HACCP Approach

Since 1906, meat and poultry processing plants have been required by congressional mandate to establish food safety and sanitation controls in their facilities, controls that are then verified by the Food Safety and Inspection Service (FSIS) of the USDA. In addition, inspectors visit slaughter facilities where they monitor the slaughtering process for hours at a time as well as visually inspect animals for potential health problems. In the past decade, calls have increased for improved technology—bioluminescent sensors that signal the presence of *Escherichia coli*, for example—to augment the work of human inspectors.

Public meat recalls are often triggered by outbreaks of disease from *E. coli* or other microbial pathogens that can affect dozens and sometimes hundreds of consumers. One notable incident occurred in late 1992 when undercooked hamburgers contaminated with the deadly O157:H7 strain of *E. coli* were sold at Jack-in-the-Box restaurants in the western United States. Resulting foodborne illness sickened hundreds of people and killed 4. According to the Centers for Disease Control and Prevention, about 20% of the implicated hamburger patties were recalled.

In 1998 the USDA implemented a national system of HACCP management for meat and poultry processing plants as a way to minimize the risk of foodborne illness and recalls. Under the HACCP system, each processor identifies the points in its operation at which health risks might occur, then takes steps to monitor and contain those risks. The hazard analysis shows that producers have identified risks linked to their production processes, explains Caroline Smith DeWaal, director of food safety at the Center for Science in the Public Interest, a Washington-based consumer rights group; the critical control points indicate where in the production process measures have been enacted to contain those hazards.

One such critical control is to periodically make a clean break in production—stopping the flow of ground meat in processing, for example, and cleaning the equipment. This helps to draw a clear line between lots of product in the event a recall is warranted. Cooking is another critical control. For instance, meatball makers cook their product at a certain temperature for a specified length of time before the next stage of production and packaging. “Pretty simple stuff,” says Smith DeWaal, who adds that most operations already had such standards in place before the implementation of HACCP; now they were just putting them on paper.

Karlease Kelly, head of the Office of Outreach, Education and Employee Training at FSIS, says her agency does not mandate specific controls or methods for inclusion in HACCP plans. Instead, plants develop plans that fit their business model. She says, “One of the primary strengths of the HACCP approach is that it allows the industry to find innovative ways to approach food safety, making plants responsible for their own systems.” All federal- and state-inspected meat and poultry processing facilities are required to implement HACCP plans for each product manufactured.

The meat industry saw other changes at the end of the twentieth century. Streamlined meat inspection methods implemented in the 1980s have reduced the number of government meat and poultry inspectors nationwide. In an article posted on its website 4 March 2008, the Washington, DC–based watchdog group OMB Watch noted that the number of inspectors at the FSIS declined by 7.5% since 1981, from about 10,000 to 9,200.

The number of slaughter facilities also shrank by about 10% over the same period, according to the FSIS, even as meat and poultry production has doubled from 52 billion pounds to 104 billion pounds in 2007. In the 12 October 2008 *New York Times Magazine*, author Michael Pollan attributed this shrinking to centralization of the meat industry, writing, “The big meat processors have been buying up local abattoirs only to close them down as they consolidate.” That means the number of FSIS employees per billion pounds of meat and poultry inspected and approved has declined by more than half since 1981 to fewer than 88 employees in 2007.

The crunch on inspector resources has, in some states, caused the FSIS to pull back on visits to smaller and more remote slaughterhouses. Joel Salatin, owner of Polyface Farm in Virginia’s Shenandoah Valley, says the nearest USDA-approved slaughter facility to his operation is an hour’s drive away. Lanou notes the incongruity of a farmer near her in Asheville who has to haul animals 2 hours away for slaughter and processing at a USDA-approved facility before bringing the meat back to sell locally. The irony, she says, lies in wanting to sell locally produced meat and finding that shipping it in from a city 3 hours away would involve less transport.

## A Fair Approach?

Small farm advocates and policy analysts are pressing for reform to address what they see as overlapping and sometimes contradictory regulations for meat producers. For example, in 36 states, state agencies implement their own inspection systems in tandem with the FSIS, whereas the other 14 states relinquish all inspections to FSIS. From Salatin’s perspective, the states that retain a hand in the inspection process tend to have a more viable, local-food-friendly network of small abattoirs than the states that don’t. He also points to examples of confusing definitions. Legally, pheasants aren’t considered poultry, so they involve no processing requirements. Likewise, there are no legal inspection requirements for wild game although they carry plenty of potential pathogens such as *Brucella*.

Pollan, writing in *The New York Times Magazine*, proposed making food safety regulations “sensitive to scale and marketplace, so that a small producer selling direct off the farm or at a farmers’ market is not regulated as onerously as a multinational food manufacturer.” He argued that food safety problems from small players are “less catastrophic and easier to manage because local food is inherently more traceable and accountable.” (There are few studies of the comparative health risks posed by small versus large processing facilities.)

From her visits to farm operations near Asheville, Lanou sees HACCP having unintended effects on small farms. “There’s a training component, an equipment component, a plan component, and monitoring,” she says. These components add up to a significant investment in staff time devoted to developing possibly multiple HACCP plans and to keeping records of the monitoring. While it’s important to have a process like HACCP, Lanou says, “I’m not sure this is the be-all and end-all system.”

“The development, maintenance, and recordkeeping of HACCP plans is much more of a resource burden on small operators because of the economies of scale,” explains Mark Schad, a former small plant owner/operator who now works with other small operations to help them attain an FSIS grant of inspection. “There is not much difference in the cost associated with a HACCP plan whether an operator makes one hundred or one hundred thousand pounds of product. Sometimes [small] plant operators are driven to make tough business decisions resulting in eliminating a product because the cost of a HACCP plan cannot be financially justified.”

As for access to inspectors, Kelly admits that some livestock operators must travel farther than they did before. She points out, however, that the FSIS must provide inspectors to any establishment with a grant of federal inspection that complies with the regulations. “If a local business determined it wanted to open a federally inspected slaughter facility in the area, it could submit a grant of inspection to the FSIS.” She says the FSIS recently issued resources to assist individuals interested in applying for a grant of federal inspection.

## Help for Small Farmers

The USDA has introduced several initiatives to help small producers with the HACCP process. The first initiative provides fact sheets, DVDs, and 2-hour educational sessions to guide small producers in designing food safety systems. A second provides resources for learning about basic sanitation, monitoring, and reporting practices as well as employee training. A third offers assistance in validating HACCP plans and practices, for example by helping farmers identify key hazards in their production operation and resources needed for monitoring the critical control points.

In terms of monitoring pathogens such as *E. coli* and *Salmonella*, Kelly says it’s within the scope of even very small operations to test themselves. “FSIS has compliance guidance on testing for generic *E. coli* and *Salmonella* created specifically for small and very small plants that has been available since the HACCP rule was implemented,” she says. “FSIS recently posted guidance for small operators on how to test for *E. coli* O157:H7.” The FSIS is also developing workshops to help small and very small plant operators use the guidance to develop testing plans that meet the needs of their specific operations.

Kelly says small farm owners “typically have access to fewer scientific resources, and through this new program, we are making every effort to provide useful assistance to them.” To that end, the FSIS has been working with the USDA Agricultural Research Service (ARS) on small-processing concerns. ARS staff test HACCP plan components (such as cooking times and drying times for jerky, for example) in facilities that mimic production practices in small and very small plants. The tests are meant to help develop best practices for research but are also shared with the FSIS and with small and very small operators, says Kelly. She describes one technology in the pipeline that will help small meat processors: a “sanitizing halo” that processors can assemble inexpensively using items from a discount superstore to deliver an antimicrobial cleanser to the surface of a carcass.

Pollan proposes that the USDA support remaining local slaughter facilities and suggests that the department create a “Local Meat-Inspectors Corps” as an expansion of a successful pilot program in Washington state in which mobile abattoirs travel among farms. “Nothing would do more to make regional, grass-fed meat fully competitive in the market with feedlot meat,” he wrote in *The New York Times Magazine*. Says Kelly, “FSIS has and will approve mobile slaughter units so long as they meet traditional sanitation [and other] requirements. Anyone interested in implementing such an operation needs to apply for a federal grant of inspection.”

The best way to streamline the HACCP process would be to reduce the actual number of plans, says Schad. “A small plant has to look for all possibilities to combine different products into the same HACCP plan,” he explains. “There are limitations to the degree of success in this approach because of the way the HACCP regulations are written and the conflicting feedback a small plant receives from FSIS.” Schad also suggests small farm operators look to extension agents from land grant universities, trade associations, and consultants for assistance.

Meanwhile, Salatin believes it is critical that farmers challenge inconsistencies in the inspection process. When a Virginia state inspector 12 years ago declared that the Polyface poultry slaughter area was unsanitary because it was not enclosed, Salatin fought that decision. A university lab conducted swab tests at Polyface and on government-inspected poultry purchased from a supermarket, and found that the supermarket birds averaged 10 times more bacteria than the Polyface samples. Salatin won the case.

The larger fight, Salatin insists, is for individual responsibility when the science and regulation are unclear. “We ought to at least let consumers have freedom of choice,” he says. “It’s not cut and dried, so we should let people take the responsibility to choose.”

## Dairy Dustup

Producers of “raw” (or unpasteurized) milk have challenged the food safety system too, although their situation differs from that of meat producers. For one thing, milk is regulated by the Food and Drug Administration, not the U.S. Department of Agriculture. A federal rule requires that milk sold for human consumption be pasteurized for interstate commerce, so the sale of raw milk is an issue at the state level.

About half the states have some type of legislation restricting the sale of raw milk. Some states let farmers sell raw milk directly to consumers or retailers, whereas others require farmers to obtain permits before selling.

According to Jessica Chittenden, director of communications for the New York Department of Agriculture and Markets, New York farmers can sell directly from their farm as long as signage makes it clear that raw milk doesn’t provide the protection of pasteurization. A proposed law would let farmers sell raw milk through retailers but would still prohibit the sale of other raw products such as butter, yogurt, and kefir, says David G. Cox, general counsel for the Farm-to-Consumer Legal Defense Fund.

The National Milk Producers Federation claims raw milk threatens public health with potentially higher levels of *Salmonella*, *E. coli*, and *Campylobacter*, three of the main causes of foodborne illness. According to the latest annual listing of foodborne illness outbreaks published by the Centers for Disease Control and Prevention, in 2006 there were 112 reported illnesses and 14 hospitalizations attributed to consumption of raw milk or cheese. Another 29 reported illnesses and 5 hospitalizations were attributed to consumption of pasteurized or unspecified milk, cheese, or ice cream. (For many outbreaks, no food was specified.)

Raw milk advocates, on the other hand, claim that pasteurization kills not only pathogens but also beneficial bacteria and proteins, nutrients that promote a stronger immune system in people who drink raw milk. However, the benefits of drinking raw milk are anecdotal and have not been studied in a systematic fashion.

In 2008 the issue came to the forefront in California, where raw milk advocates pushed for relaxation of the standards for coliform bacteria in milk, saying the federal standard of 10 bacteria per milliliter pasteurized milk does not take into account healthy bacteria that remain in raw milk. Instead, raw milk proponents recommended using producers’ HACCP plans coupled with testing for pathogens to oversee health safety—review of a farm’s HACCP plan and the data the farm submits on its monitoring process would be the basis for identifying critical points, not inspection. State Senate Bill 201 would also have required dairies to be tested monthly for *E. coli*, *Salmonella*, and other pathogens, which Cox says is not required under existing California law for either raw or pasteurized milk producers.

The California legislature passed the bill, but governor Arnold Schwarzenegger vetoed the bill on 30 September 2008, calling it “convoluted and undefined regulatory process with no enforcement authority or clear standards to protect public health.”

## Figures and Tables

**Figure:**
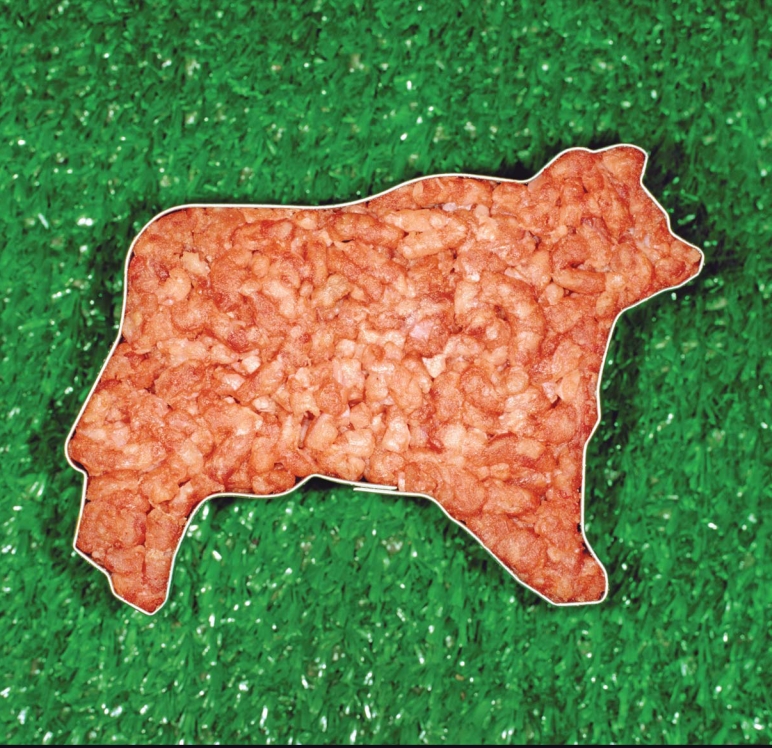


**Figure:**